# ‘They’re creepy creatures with human-like features’: children’s experiences of visual hallucinations in Charles Bonnet syndrome—a qualitative study

**DOI:** 10.1136/archdischild-2024-327811

**Published:** 2025-01-09

**Authors:** Lee Jones, Lara Ditzel-Finn, Leanne McDonald, Mariya Moosajee

**Affiliations:** 1 Institute of Ophthalmology, University College London, London, UK; 2 Great Ormond Street Hospital for Children NHS Foundation Trust, London, UK; 3 Department of Psychology, University of West London, London, UK; 4 Moorfields Eye Hospital NHS Foundation Trust, London, UK; 5 The Francis Crick Institute, London, UK

**Keywords:** Child Health, Child Psychiatry, Mental health, Ophthalmology, Qualitative research

## Abstract

**ABSTRACT:**

**Objective:**

Charles Bonnet syndrome (CBS) refers to the presence of visual hallucinations occurring secondary to visual impairment. The aim of this study was to understand the phenomenology of CBS in children and assess the emotional impact and support needs of patients and their families.

**Design:**

Semistructured qualitative interview study.

**Setting:**

UK.

**Participants:**

Children (7–15 years) with an inherited retinal disease living with CBS and their parents.

**Results:**

10 participants were recruited from six families (dyadic interviews n=4; parent-only interviews n=2). Thematic analysis identified five superordinate themes relating to experiences of CBS: (1) diagnosis journey, (2) hallucination phenomenology, (3) impact of hallucinations, (4) understanding and managing hallucinations and (5) experiences of support. The impact of CBS was broad and heterogenous, causing significant disruption to patients’ daily life. Limited awareness led to parents expressing largely negative healthcare experiences. Overall, the extent of knowledge and understanding of CBS was an indicator of successful self-management of the condition.

**Conclusions:**

The journey towards understanding and managing CBS for both parents and children is challenging. Although coping strategies can lead to improved adjustment, visual hallucinations compounded the difficulty of living with a chronic visual impairment. Healthcare providers have an integral role in ensuring patients and families are effectively supported to allay fears and promote psychological well-being.

**WHAT IS ALREADY KNOWN ON THIS TOPIC**

- Charles Bonnet syndrome (CBS) occurs secondary to sight loss.
- Research into children’s experiences of the condition is sparse, although there are concerns visual hallucinations in this cohort have potential to cause considerable upset/psychological harm.

**WHAT THIS STUDY ADDS**

- CBS caused significant disruption to daily life.
- Limited understanding of the condition among the public and healthcare professionals hinders appropriate clinical management decisions, such as referral to CBS patient support.
- Receiving an accurate diagnosis was reassuring for patients and their families and led to improved psychological adjustment.

**HOW THIS STUDY MIGHT AFFECT RESEARCH, PRACTICE OR POLICY**

- Increased awareness of CBS occurring across all ages is necessary to ensure patients and families are equipped to live well with the condition.

## Introduction

 Charles Bonnet syndrome (CBS) is a condition where visual hallucinations occur secondary to visual impairment (VI). The underlying pathophysiology is generally considered to be deafferentation, whereby cortical hyperexcitability following reduced input to the visual cortex causes spontaneous visual hallucinations.^1^ Typical phenomenology includes transient episodes of simple or complex hallucinations that are purely visual, with no other affected modalities. Simple hallucinations consist of geometric shapes and patterns (ie, tessellopsia), colours, lights and flashes (ie, photopsia), whereas complex hallucinations involve formed images including people, animals and vivid scenery.^2 3^ Prevalence of CBS has been estimated at 10–20% of adults with VI^4^; however, these estimates vary widely.^5 6^ Individuals with more advanced vision loss have an elevated risk^7^; however, the condition also occurs in patients with largely preserved vision.^8 9^

Despite patients of any age being at risk, most research has been directed towards older adults. This may be explained by age being a risk factor for many sight-threatening conditions. Yet, a small number of studies describe the phenomena occurring in children and adolescents, reporting the clinical profiles of those affected.^10^,^12^ These studies provide initial insights into CBS in young people, however, are limited in elucidating patients’ experience of the condition. This is significant given that CBS in adults may cause considerable anxiety and distress.^13^ For example, hallucinations are often macabre, such as faces with a grotesque or distorted appearance (ie, prosopometamorphopsia) or include ominous cloaked or uniformed figures.^14^ Retaining an understanding that visual hallucinations are not real can have a protective effect on psychological well-being; however, children may have a tendency to believe their hallucinations are real,^15^ suggesting this cohort may be more susceptible to maladaptive responses to hallucinations, such as fear and distress.

Understanding young people’s experiences of CBS is essential to enable clinical and research priorities to be mapped against the needs of patients and their families. The aim of this study was to explore the experience of CBS from the perspectives of children and their families, including hallucination phenomenology, emotional impact and accessing support.

## Methods

The study used semistructured interviews with parent and/or children with CBS. All parents and children were told about the study aims and provided written consent to take part. The study was designed and reported following the guidance of the Consolidated Criteria for Reporting Qualitative Research.^16^

Purposive sampling was used, whereby participants were recruited from Moorfields Eye Hospital and Esme’s Umbrella, a charity for people living with CBS. Eligible participants were between the ages of 5 and 16 years and diagnosed with CBS. Recruitment advertisements were published via social media and participants were screened for CBS via a detailed patient history prior to inclusion. Dyadic interviews were conducted online, where the child and parent were interviewed together. The topic guide was developed with input from Esme’s Umbrella. Interviews were conducted by a female specialising in child and adolescent health psychology (LD-F), with a personal interest in CBS and no prior relationship with participants. Six families were recruited with 10 participants interviewed (dyadic interviews n=4; parent-only interviews n=2). No participants withdrew from the study. Parent-only interviews were conducted as requested by participants. A summary of participant demographics is provided in online supplemental table 1.

Average interview duration was 40 min. Audio-recorded interviews were transcribed verbatim. Transcripts were analysed using thematic analysis,^17^ whereby texts were read and meaningful units were coded. Data were coded by two researchers (LJ, LM) and discussed among the entire research team. Data saturation was defined as when no new information was coded during transcript analysis, indicating further data collection or analysis was unnecessary. After coding, themes were generated by identifying patterns across interviews and collecting relevant codes together. Themes were created inductively (ie, driven by the content of the data) due to the limited previous research in the area, and therefore no specific theories were used to generate themes. Data were analysed using NVivo V.13 (QSR International, Cambridge, Massachusetts, USA).

## Results

Average age of the children at the time of interview was 11±2.9 years. Five patients were diagnosed with an inherited retinal dystrophy (IRD) and one with a hereditary optic neuropathy. IRDs are a group of genetically and phenotypically heterogenous conditions affecting 1:1000 people worldwide.^18^ Analysis revealed five superordinate themes related to experiences of CBS: (1) diagnosis journey, (2) hallucination phenomenology, (3) impact of hallucinations, (4) understanding and managing hallucinations and (5) experiences of support.

### Theme 1: diagnosis journey

A common theme was the widespread lack of awareness about CBS among both parents and healthcare professionals. Parents described how they were initially unaware of CBS prior to their child receiving a diagnosis. Many parents initially dismissed their child’s visual hallucinations as imaginary friends or typical childhood experiences. When reporting symptoms to clinicians, participants encountered a similar lack of awareness, which became a source of frustration. Even where professionals recognised the symptoms of CBS, there was uncertainty and caution applying a definitive diagnosis. This led parents to prioritise their own research, seeking information online to better understand the condition. Once aware, this largely alleviated concerns and normalised the condition. Limited awareness of CBS resulted in delays in receiving an accurate diagnosis, leaving parents feeling unsupported despite their proactive efforts to educate themselves. Diagnosis journey subthemes and example participant quotes are provided in table 1.

**Table 1:** Diagnosis journey subthemes with participant quotes

Theme 1: diagnosis journey	Quote
Parents’ awareness	‘I didn’t understand what it meant. It’s almost as if it was a myth. That’s the way I viewed it when I first started researching it. I thought, is this actually a thing?’ (P1)
Parents’ dismissal of symptoms	‘I had Christmas wrapping on the floor and she said she could see a face in the wrapping paper. I thought she was imagining things.’ (P2)‘She’d say, Mummy, I’ve seen a dog and it was in the air. I didn’t believe her, because she was the age she was.’ (P5)
Clinician awareness	‘The doctor said you don’t normally get hallucinations in sight loss and that you only normally get that in people with Alzheimer’s. I think that’s shocking that doctors in their own fields don’t know it. That’s quite sad.’ (P6)‘The optometrist that was seeing her had never heard of it in children. She’d heard of it in adults but had never heard of it before in children.’ (P4)
Diagnostic uncertainty	‘Her optometrist had heard of it, he thought it was maybe CBS but he didn’t want to say because he wasn’t 100 per cent sure.’ (P2)
Parents’ research	‘I’d come across Charles Bonnet syndrome when I was Googling it. I was thinking this could be related to his eyes because it would make sense. Reaching out to other people within the community, they have said the same thing.’ (P1)
Receiving an accurate diagnosis	‘When the doctor told me a bit more about it and then I investigated a little bit more about it, I kind of felt it’s like a routine now, so I kind of expect it.’ (C2)

CBS, Charles Bonnet syndrome.

### Theme 2: hallucination phenomenology

Children reported both simple and complex hallucinations as part of their experiences. Simple hallucinations were typically colourful shapes, although descriptions varied among participants. One participant described experiencing only complex hallucinations, whereas they had previously encountered simple ones, suggesting the nature of their hallucinations had changed over time. This participant attributed this change to a deterioration in their vision, a belief echoed by others in the study. Complex hallucinations were frequently described as scary or frightening, taking the form of figures or entities. In some instances, these hallucinations resembled recognisable characters, but with distortions that rendered them distressing. Additionally, many participants reported hallucinations involving insects, such as spiders, flies and wasps. Hallucination phenomenology subthemes and example participant quotes are provided in table 2.

**Table 2:** Hallucination phenomenology subthemes with participant quotes

Theme 2: hallucination phenomenology	Quote
Simple hallucinations	‘I just see colours and it blocks my vision, so I couldn’t really see what’s in front of me. It’s a bit scary.’ (C2)‘He says he sees lots of zigzags and circles, and different shapes.’ (P1)‘I could see like patterns of zebras all around my room.’ (C5)
Complex hallucinations	‘It started with just flickering and I was completely fine because it barely caught my attention, but then went away quite suddenly. But nowadays, it’s quite frightening. What I experience is things, like entities, a bit like they’re creepy creatures with human-like features. I’m not quite sure how to describe it really. They’re like literal beings as if they’re actually there. It has human features and it would move. It’s not a still image when I see it. It would actually move from side to side. It could hide behind a door. It could just vanish. They’re like modern-day horror movie entities.’ (C6)‘It’s like those mannequin faces that you see in the shops, those kinds of faces—it’s quite like scary.’ (C2)
Character-related hallucinations	‘He woke up in the middle of the night and he could see Peter Rabbit in the bedroom with him, but like a scary version of Peter Rabbit.’ (P1)‘It’s a bit like—have you ever watched Harry Potter—those black things that fly in the sky. It’s like those dementors.’ (P6)
Insect-related hallucinations	‘One night it was just horrendous where he thought there was a wasp on his face, and he was so adamant that there was a wasp on his face. He kept trying to smack it off.’ (P1)‘I see spiders now. They used to be big but now they’re usually small. Sometimes they can still be big. They can be massive spiders and then there’s very tiny things, tonnes of them.’ (C4)‘He was adamant he could see red spiders all over the rug.’ (P1)
Changing hallucinations with worsening vision	‘They started quite small. Since my blind spot was quite small at that time, it wasn’t really that strong or I didn’t really notice it too much when I first got it. But over the years since my blind spot is growing more and more, it’s growing as well as I get older. The hallucinations have been rapidly increasing in times it has been appearing, and also how much it affects me.’ (C6)‘The pictures change and she’s starting to get a lot more complex shapes now. She describes brick walls, stuff like that. It’s just hard and really, really taking an emotional and physical toll on her now.’ (P3)

### Theme 3: impact of hallucinations

CBS had a profound and multifaceted impact on day-to-day life, affecting emotional, social and physical well-being. Children experienced a range of emotions, with fear being the most frequent, often linked to the distressing nature of the hallucinations and the unpredictability of their onset. Agitation sometimes prevented children from feeling comfortable enough to return to locations where they had previously encountered hallucinations. One parent explained how their child felt unable to visit a grandparent’s house due to such fears. Emotional responses varied depending on the nature of the hallucinations, including their proximity and content. For most, CBS caused significant issues with sleeping and bedtime. While school life was generally not affected, one participant reported hallucinations interfered with their ability to complete schoolwork. Hallucinations compromised physical safety, with participants feeling more prone to falls, accidents or risky behaviours due to their vision being obscured or their fear of the images. In some cases, this led participants to avoid certain activities altogether, which in turn resulted in isolation from social networks. One participant expressed difficulties explaining CBS to friends, further highlighting the social challenges posed by the condition. Impact of hallucination subthemes and example participant quotes are provided in table 3.

**Table 3:** Impact of hallucination subthemes with participant quotes

Theme 3: impact of hallucinations	Quote
Fear and distress	‘He’s normally just quite scared, I don’t think he really understands. At times I’d say he’s beside himself. He’s completely and utterly freaked out.’ (P1)‘I get quite panicked and get an urge to just shut my eyes. But I know that won’t help because it’ll still be there even if I shut my eyes. It kind of pops up and there’s no warning of when it does. It’s scary because it just comes and goes, kind of like as it pleases.’ (C2)‘I was dancing outside, I thought I saw someone pop out behind the wall, and then I was just too scared to go over there. It just scared me too much that I couldn’t walk that way anymore.’ (C5)
Impact on routine activities	‘He saw a witch on the staircase looking up the stairs at him. His gran had kitted out all of her spare room for him with all of his toys and his bed in there. After that happened he would not even go in there. To the day that she died he would not go there—he was so disturbed by what he’d seen in that room.’ (P1)
Impact on sleep	‘She’s not sleeping very well at all because of her hallucinations now, the doctor has actually given us something to help her sleep and she’s been referred to a psychiatrist for further support because she’s really struggling.’ (P3)‘He would be in his bedroom, and he’d be screaming because he wouldn’t be able to get up. He wouldn’t be able to leave the room because all these things had come around him.’ (P6)
Impact on schoolwork	‘When I’m sitting doing my homework and I’m like almost done and then it just pops up and then I can’t finish it because I can’t see what I’m doing. That annoys me—well it makes me quite angry because one minute I’m doing something like reading and the next minute I can’t see anymore.’ (C2)
Impact on physical safety	‘She got an image and she fell so badly in the alleyway that she hurt herself.’ (P3)‘I came home from school, but it was in winter so it was pitch black by four o’clock. When I came back—it frightened me so much that I actually ran home without looking back for anything. I just ran across roads as well. It can influence my life quite significantly sometimes.’ (C6)
Impact on social activities	‘She’s almost like a recluse in the evenings because she’s very conscious of people knowing about her condition.’ (P3)‘I can explain it to people but I have to think how am I going to explain this to somebody who’s never had it before or possibly never is going to have it. So that’s why I’m a little bit nervous. I think oh how am I going to explain this?’ (C2)
Nature and proximity of hallucinations	‘Sometimes it can just be kind of weird feeling, and then some of them can be scary, like, if I see like a face, that would scare me.’ (C5)‘If it’s further away I’m less frightened of it. But if it’s in my house and really close to me, that’s quite scary, that’s quite frightening.’ (C6)

### Theme 4: understanding and managing hallucinations

Several potential triggers were reported, including low lighting, stress and stimuli from the physical environment. Among these, low lighting—particularly at night—was the most common. Participants described how hallucinations could emerge from around corners, such as doors and doorframes. One participant shared a strategy of changing the physical environment, such as closing doors to avoid potentially triggering situations. Stress was widely regarded as a contributing factor, with participants developing strategies to manage their symptoms. Strategies included making changes to their physical surroundings and using head or eye movements to disrupt or displace the hallucinations. Participants reported success with several coping mechanisms, including self-regulation techniques like breathing exercises and self-soothing behaviours. A common approach was to remind themselves that the hallucinations were not real. Even among younger participants, maintaining insight was a crucial factor in managing anxiety. Understanding and managing hallucination subthemes and example participant quotes are provided in table 4.

**Table 4:** Understanding and managing hallucination subthemes with participant quotes

Theme 4: understanding and managing hallucinations	Quote
Onset in low lighting	‘They annoy her, especially at night. She says Mummy, I don’t get many throughout the day, but I get loads at night.’ (P3)‘It’s quite random. I see them whenever I’m in a dark place usually. That’s when I see them, but normally they’re just random really.’ (C4)
Onset during stress	‘Definitely stress is playing a huge part and if we keep her stress-free, she doesn’t have them as much, but when she’s stressing more, then they are far more frequent.’ (P3)‘I can ignore it more when I’m distracted. I think when I’m stressed or upset it does happen more.’ (C6)
Triggers within physical environment	‘I was in my bedroom, and it was really dark, and then I could see a figure standing by my door. My door was closed and I knew that it wasn’t there, but it was even scarier than it would probably be in daylight.’ (C5)‘Some of the triggers are, for example, if I’m doing a load of ironing and I hang them on the doorframe, I have to warn him in advance because he comes along and he sees headless bodies or people hanging.’ (P6)
Strategies to mitigate hallucinations	Changing physical environment: ‘If I close off a door, it shuts off more vision of what I can see, so it reduces the amount of what could happen. There’s nothing that could appear out of thin air behind anything.’ (C6)Blinking: ‘There are some nights where there’s smaller ones which don’t really bother her because she can move them by blinking her eyes, she can move them to the corner.’ (P3)Eye and head movements: ‘I just look away, when I look away, and then look back, most of the time, they’re gone.’ (C5)Self-regulation: ‘Sometimes I just take a minute and just breathe. I calm myself down, or I just take a minute just to get calm again.’ (C2)Reframing: ‘I’ve learned to understand that they’ll just be there, sometimes, and then I know that mostly, I just know that they’re not real, or it’s not there.’ (C5)Reframing/changing physical environment/touching the hallucination: ‘I talk to myself to say that they’re not real. I turn the light on to see if that helps. Sometimes I get mum or dad. Sometimes I try to picture them like they’re cute little things instead of scary things. You try to touch them to prove they’re not there.’ (C4)

### Theme 5: experiences of support

Parents generally agreed that their families had not been sufficiently supported. In addition to the perceived lack of knowledge among clinicians, the information provided often failed to meet their expectations or answer their specific questions. Some participants highlighted the value of accessing specialist support services, such as Esme’s Umbrella, which offered an understanding of CBS. Despite this, there was a desire for enhanced support systems, such as peer support. However, participants reported difficulties in finding other young people with lived experience of the condition. Overall, there was a shared desire among participants to assist others with CBS, with the hope that participating in research would raise awareness about the need for improved support pathways and more effective management strategies to help future patients. Experiences of support subthemes and example participant quotes are provided in table 5.

**Table 5:** Experiences of support subthemes with participant quotes

Theme 5: experiences of support	Quote
Frustrations with limited support	‘Literally all what I know about it is in a leaflet, that’s it. That’s all I knew about it.’ (P2)‘As a parent, there’s key questions that I want answered, I want to know how long is she going to be like this, how long is it going to last, what can I do, will this just go away? I’ve realised that I’m not going to get answers to my questions because nobody knows. Then how am I supposed to reassure her when I don’t even know the answers myself?’ (P3)‘When he was diagnosed, we got a leaflet. There was a leaflet on Charles Bonnet, which I looked into. That’s the only information I ever got. I never got anything else. It was down to me to do all the research then.’ (P6)
Specialist support services	‘I spoke to Esme’s Umbrella, they told me it’s quite normal for people with CBS to see things.’ (P3)
Peer support	‘The charity wanted to find someone she could speak to who’s got something similar so they can bounce ideas off each other, but the charity didn’t know anyone.’ (P3)
Desire to help others	‘My advice to stop it is to change the atmosphere of where you are to suit you. Change the setting to suit your needs, to make sure you’re comfortable. If you’re uncomfortable, stressed or just really upset at that point in time, it increases how many times you see these hallucinations.’ (C6)‘It would have been better if I’d known somebody who had it so we could chat about it and what strategies we use to help in the situation.’ (C2)‘I was very keen to do this study just to help other children because it has been a scary time for her, especially when we didn’t know what was happening or what it was. It’s been reassuring for her just to know what this condition is and why she’s having these hallucinations. She was keen just to be able to help other children.’ (P4)

CBS, Charles Bonnet syndrome.

## Discussion

This qualitative study provides an in-depth exploration of children and their families’ experience of living with CBS. Overall, participants described mostly negative experiences, where hallucinations had caused considerable upset with long-standing residual effects. Perspectives on the quality of healthcare and support that had been received were largely considered suboptimal.

Although hallucinations have previously been researched in children with clinical history, such as schizophrenia,^19^ and emotional disorders,^20^ little is known about CBS among children. Studies have identified that children experience distress and impaired functioning as a consequence of hallucinators; however, research has generally focused on auditory hallucinations, such as hearing voices.^21^ Few studies address visual hallucinations in children; however, among these, there is consensus that visual hallucinations evoke anxiety.^12^ Previous research suggests complex hallucinations have a strong influence on children’s mental health, significantly affecting levels of depression, anxiety and dissociation.^22^ Families in our study described a significant negative impact of CBS, suggesting more efforts are needed to facilitate a shared understanding of young people’s experience, which can help inform the development, implementation and evaluation of support pathways to improve healthcare services.

Recent recommendations outline the clinical management for patients with visual hallucinations in high-burden areas, including CBS.^23^ The consensus framework was developed following the Study of Hallucinations in Parkinson’s Disease, Eye Disease and Dementia (SHAPED) trial, which suggests management should begin prior to the onset of hallucinations, through routine provision of information at the time of consultation, informing patients of their susceptibility, and pre-emptive questioning to encourage reporting. Participants in our study reported being unaware of CBS prior to symptoms presenting, and expressed frustration at the lack of information they were provided. These findings align with others demonstrating low awareness of CBS among patients and healthcare professionals, particularly those with less specialist experience in ophthalmology.^24 25^ Despite recommendations to forewarn patients, the extent to which these conversations are facilitated in practice is unclear. Taking together our findings and the SHAPED recommendations,^23^ ophthalmologists, paediatricians and all those working with children diagnosed with progressive or severe sight loss should be encouraged to ask and inform patients about visual hallucinations.

Superficial commonalities in the phenomenology of CBS between adults and young people should not obscure potentially significant differences between these groups. For example, clinically significant CBS, in other words, patients who experience distress despite reassurance, accounts for around one-third of all cases.^14^ Yet, all families in this study reported experiencing distressing hallucinations. In that respect, children may be more susceptible to clinically significant CBS, imposing a negative effect on psychological well-being. This may be compounded by younger people having a diminished ability to distinguish between hallucinations and real life^15^; however, participants in this study generally recognised hallucinations from reality. These findings represent diagnostic challenges in CBS case ascertainment among children. For example, CBS may be confused with childhood imaginings, and children may have difficulties describing their symptoms.^15^ Imaginary friends are common among children but differ from visual hallucinations, such as they are often invoked by the child at will and typically function as a play partner associated with positive emotions.^26^ A further challenge is that visual hallucinations have a broad differential diagnosis and may initially be investigated as schizophrenia/schizoaffective disorder, migraines, seizures or tumours.^27^

Our results provide qualitative details regarding ophthalmological associations in CBS. Previous studies report better eye visual acuity of Snellen 6/18 or worse as a threshold for increased risk of CBS.^7^ Similar studies suggest CBS occurs more frequently among groups with more extensive VI.^28^,^30^ A participant in our study reported changes in the phenomenology of their hallucinations corresponding to the worsening of their blind spot (ie, visual field scotoma). The participants explained: *‘over the years since my blind spot is growing more and more, it’s growing as well as I get older. The hallucinations have been rapidly increasing in times it has been appearing.’* This finding supports an association between the degree of VI and CBS. Yet, visual acuity is not an accurate metric to inform CBS risk as signals from the eye can be reduced without impacting acuity.^31^ Our previous work has identified CBS in children with low vision in one eye but normal vision in the contralateral eye, as well as in those with non-progressive congenital eye defects.^12^ Several studies have concluded that poor visual acuity is not a primary risk factor for CBS.^8 9^ Other theories suggest hallucination content reflects the functional specialisation of the activated region of the brain. For example, hallucinations of colours, faces, textures and objects correlate with cerebral activity in the ventral extrastriate visual cortex. Specifically, textures such as brickwork are linked with activity around the collateral sulcus and hallucinations of faces and objects are associated with activity in the left middle fusiform gyrus and right middle fusiform gyrus, respectively.^32^

Several potential triggers were reported including low lighting, specific stimuli and stress. Visual hallucinations occur predominantly under low-lighting conditions as brightness aids object perception, while darkness removes cues to activate scene representation. Research in Parkinson’s disease identifies ambient lighting as an important factor for the onset of visual hallucinations.^33^ The specific role of lighting in CBS remains unclear as patients report experiencing hallucinations in bright light, dim light and darkness. Participants described how seemingly mundane and innocuous stimuli could lead to distressing hallucinations, such as laundry resting on hangers becoming ghost-like figures, and faces emerging in gift wrapping paper. These examples could be explained by visual perception theories which implicate impaired attentional binding and poor sensory activation of an object, biasing perceptions to allow the intrusion of a hallucinatory object into a scene perception.^34^ These stimuli may be perceived as triggers as once an image is hallucinated, it may become associated with a specific hallucinatory scene representation, increasing the probability of the same image being triggered again and may account for the repetition of specific images.^34^ Stress was widely believed to be a contributing factor, and studies suggest stressful life events, such as natural disasters and pandemics, may trigger more clinically significant CBS.^35 36^ Parents were worried about their child’s mental health and often strived to minimise stress to reduce hallucinations. Anxiety symptoms and disorders are common problems associated with patients with ophthalmic disease, including child and adolescent patients.^37^ Notably, patients with CBS report worse scores on measures of anxiety, depression and social dysfunction when compared with patients with VI without CBS,^38^ suggesting children with CBS are at an elevated risk of experiencing mental health problems.

A range of management strategies were described, including changes to the physical environment, such as increasing room brightness, and practising specific head or eye movements, or repetitive blinking. Anecdotal evidence suggests these ‘brain shunting’ exercises can be effective in disrupting CBS.^39^ These behavioural strategies can be recommended to all patients to equip them with skills to mitigate the impact of visual hallucinations, although more research is required to establish a robust evidence base to better understand their therapeutic effects. In cases where CBS causes significant distress, referral to child psychiatry for formal assessment and management may be required. Psychological therapies such as cognitive–behavioural therapy or pharmacological treatment may provide relief,^40^ though a robust evidence base regarding the long-term efficacy in both children and adults is lacking.

This study is limited in that a small number of families were interviewed and they were all based in the UK, which may restrict the transferability of our findings. Moreover, our use of parent–child dyadic interviews is arguably a limitation as this may have influenced responses through social desirability; yet, we believe this was also a strength as it encouraged expression among the children which increased the richness of the data.

In summary, our results provide new evidence relating to children’s experiences of CBS, and visual hallucinations more broadly. These reflections draw attention to priority areas for clinical service improvements, such as greater knowledge and awareness and better signposting to support. Despite the availability of online resources, most felt they had not been provided with sufficient support and information, resulting in participants doing their own research. The research team have worked with Esme’s Umbrella to provide an online resource with information about CBS targeted towards children and young people (www.charlesbonnetsyndrome.uk/children-young-people). An increased understanding of CBS appeared to be an indicator of better adjustment and psychological health outcomes; thus, screening children and young people to obtain the relevant history about CBS has potential to yield large benefits for patients and their families.

## Supplementary material

10.1136/archdischild-2024-327811 online supplemental file 1

## Data Availability

No data are available.
