# Supplementary material for: ‘They’re creepy creatures with human-like features’: children’s experiences of visual hallucinations in Charles Bonnet syndrome—a qualitative study
Source: Arch Dis Child. 2025 Jan 9;110(5):e327811. doi: 10.1136/archdischild-2024-327811 (PMC12013589; doi:10.1136/archdischild-2024-327811)
Supplement: online supplemental file 1 [file archdischild-110-5-s001.pdf]

Participant	Sex of child	Diagnosis	Child age at interview (years)	Age at CBS onset (years)
P1 (parent only)	M	Retinal dystrophy	7	2.5
P2/C2	F	Rod-cone dystrophy	13	7
P3 (parent only)	F	Retinitis pigmentosa	14	10
P4/C4	F	Retinitis pigmentosa	11	8-9
P5/C5	F	Hereditary optic neuropathy	11	10-11
P6/C6	M	Stargardt disease	15	8

Supplementary Table 1. Participant demographics. Key: P = parent; C = child.
